# Adsorption of cationic and anionic dyes using chemically modified activated carbon from agricultural waste

**DOI:** 10.1038/s41598-026-59532-5

**Published:** 2026-07-04

**Authors:** Toka M. Bahr, Ahmed M. Hassan, Mohamed El Saied, Khaled M. Abdelbary

**Affiliations:** 1 Agricultural Engineering Department, Faculty of Agriculture, Cairo University, Giza, Egypt; 2 Egyptian Petroleum Research Institute, Cairo, Egypt

**Keywords:** Agricultural waste, Activated carbon, Methylene blue, Congo red, Adsorption, Kinetic and isotherm, Chemistry, Environmental sciences, Materials science

## Abstract

Methylene blue (MB), a cationic dye, and Congo red (CR), an anionic dye, were adsorbed from aqueous solution using activated carbons that were prepared from date pits (date pits AC) biomass. These adsorbents’ surface chemistry was described using a variety of analytical methods, and the corresponding adsorption interactions were examined using the findings. The adsorption mechanism was discussed based on adsorption isotherm, kinetic, and surface characterization results. In order to assess the adsorption behaviour of the biomass, batch adsorption studies were carried out and a number of parameters, including pH, initial concentration of the adsorbates, adsorbent dose, time, and temperature, were optimized. Results showed that the maximal uptakes of MB were 833 mg g^−1^ at pH between 5.5 to 10 and 743 mg g^−1^ for MB and CR at pH between 2 and 4.5 respectively. The pseudo-second-order model provided the best fit to the kinetic data, indicating that chemisorption may play a role in the adsorption process for both MB and CR dyes. The findings demonstrated that the inexpensive biomass-derived adsorbent has the potential to effectively remove both cationic and anionic dyes from wastewater.

## Introduction

Waste that is derived from biomass comes from a variety of sources worldwide. By submitting to pyrolysis units, biomass wastes can be utilized as a sustainable source of bioenergy^1^. However, there have recently been calls for an alternative use of biomass waste, such as the creation of composite materials and activated carbon (AC)^2^. One of the most environmentally beneficial approaches is the production of activated carbons^3^. Because of their high specific surface area (up to 3000 m^2^ g^−1^) and highly developed porosity, activated carbons (ACs) are renowned as very effective adsorbents^4^. Furthermore, ACs can offer a high degree of surface reactivity because their surface chemistry is typically tunable^5^. ACs are promising materials for a variety of applications beyond just adsorbents because of these special qualities. They can also be used as catalysts and catalyst supports, among other distinct applications^6^. The Arecaceae family, also known as Palmae, includes palms, which are extensively cultivated in tropical and subtropical regions^6^. The date palm (*Phoenix dactylifera L.*) is a member of the Arecaceae family, which comprises over 2000 species and more than 150 genera. The date palm fruit seed, which makes up about one-fifth of its total weight, is primarily composed of protein, carbohydrates, fibre, fat, oil, minerals, and other vital nutrients. Some other names for the seed include pit, kernel, and stone. In comparison to the other parts of the tree, date palm seed has superior physicochemical qualities and is the primary source of biomass^7^. Numerous studies have been carried out to prepare and use biochar and activated carbon (AC) using various date palm tree parts, primarily through pyrolysis and hydrothermal carbonization. In addition to crystallinity, morphology, functional groups, pore distribution, and surface area, batch adsorption, photocatalysis, and antibiological processes were used to characterize the optical, catalytic, and biological activities of AC and biochar. Date palm seeds demonstrated exceptional physicochemical qualities that demonstrated superior performance in a variety of applications, according to numerous published studies.

There are various ways to produce activated carbons^7^. According to the activation process, these techniques can be broadly categorized into two groups: chemical and physical activation^8^. The primary goal of the activation process is to enhance the porosity and total pore volume of the generated activated carbon, as well as to control the diameter of the pores^9^. In the physical activation process, a precursor is carbonized in an inert atmosphere at high temperatures (500–900 °C). After the carbonization process, the resultant char is activated at high temperatures (800–1000 °C) in the presence of steam or CO_2_^10^. On the other hand, the chemical activation process involves impregnating raw material with an activating reagent, followed by heating the impregnated material in an inert atmosphere. In this approach, the activation process and the carbonization step occur simultaneously^11^. By using a chemical agent, the activation process is carried out at temperatures between 500 and 700 °C, which enhances the formation of pores in the final activated carbon structure^12^.

A variety of substances have been used as chemical activating agents, including ZnCl_2_, H_3_PO_4_, NaOH, and KOH^13^. These agents share characteristics, such as dehydration and degradation, which allow them to increase the porosity of carbonaceous materials^14^. They typically affect the decomposition of solid waste and prevent tar formation, resulting in a significantly higher yield of activated carbon^15^. The following succinctly describes the benefits of the chemical activation method over the physical one: it yields a significantly higher yield and requires lower operating temperatures. The chemical activation can be completed in a single step, in contrast to the physical one. Another benefit of using the chemical process instead of the physical one is that activated carbons have high specific surface areas. Lastly, the porosity of the activated carbon obtained via the chemical activation approach may be precisely regulated and kept within a small range^16^.

Adsorption is considered the most efficient way to treat wastewater since it is inexpensive, simple to use, effective, and environmentally friendly^17^. Therefore, activated carbon derived from biomass is considered one of the most important materials for removingsynthetic dyes. Synthetic organic dyes are widely used for colouring in industries, such as textile, paint, printing, paper, art, biotechnology, and rubber^18^. These organic dyes contain specific chromophores, which are primarily basic and acidic functional groups. In particular, sulfonic or carboxylic acid groups are present in acidic colours, whereas amino groups are present in basic dyes^19^. For both humans and aquatic life, these pigments are extremely poisonous, mutagenic, and carcinogenic^20^. Furthermore, they can change the ecological balance by reducing light transmission and influencing the process of photosynthesis. According to the Ecological and Toxicological Association of Dye Stuffs Manufacturers (ETAD), 90% of the dyes used were extremely poisonous, with LD50 values much greater than 2 × 10^3^ mg kg^−1^^21^. Congo red (CR) is a typical anionic diazo dye that is frequently used in textiles, lab experiments, and other commercial products, while basic methylene blue (MB) is used in the cotton, silk, paper, and ink industries due to its unmatched colour stability and water solubility. In addition to causing respiratory irritation, diarrhea, vomiting, dizziness, mucous membrane damage, and genetic mutations, the toxicity of MB and CR dyes damages the nervous system^22^.In this work activated carbon was prepared from agriculture waste, date pits,using KOH and ZnCl_2_. The performance of the activated carbon in removing both methylene blue and Congo red as models of anionic and cationic dyes was evaluated. Several factors affecting the adsorption process were studied, including temperature, pH, contact time, initial dye concentration, adsorbent dosage, and the AC reusability over multiple cycles.

Despite the extensive research on activated carbon production from various agricultural residues, limited attention has been given to date pits as a region-specific and abundantly available biomass precursor in Middle Eastern countries. The Middle East represents one of the largest date-producing regions worldwide, generating substantial quantities of date pits waste annually. The disposal of this biomass often poses environmental and management challenges. Therefore, valorizing date pits into high-value activated carbon not only contributes to sustainable waste management but also supports circular economy strategies in the region.

Moreover, although chemical activation using different agents has been widely explored, a systematic evaluation of ZnCl_2_ activation for optimizing the structural and adsorption properties of date pit-derived activated carbon remains insufficiently addressed. In this context, the present study aims to fill this gap by developing and optimizing ZnCl₂-activated carbon from date pits and investigating its structural characteristics and adsorption performance. The findings provide a sustainable, high-efficiency adsorbent derived from regionally abundant biomass, thereby contributing both environmentally and scientifically to the existing literature.

## Experimental

### Materials

In this investigation, potassium hydroxide (KOH), zinc chloride (ZnCl_2_), hydrochloric acid (HCl), and sodium boron hydride (NaBH_4_) were bought from Sigma-Aldrich in the UK and used exactly as supplied, without any additional processing. Deionized water was used throughout the experiment. Congo red (CR) and methylene blue (MB) were purchased from Lab-scan in Thailand. The physical and chemical properties of MB and CR are shown in Table 1.

**Table 1:** The primary physical–chemical properties of Congo red and methylene blue.

	Methylene Blue	Congo Red
Chemical structure		
Chemical name	[7-(dimethylamino)phenothiazine-3-ylidene]-dimethylazanium;chloride	Disodium;4-amino-3-[4-[4-[(1-amino-4-sulfonatonaphthalen-2-yl)diazenyl]phenyl]phenyl]diazenyl]naphthalene^−1^-sulphonate
Dye category	Cationic mono-azo dye	Anionic di-azo dye
Melting point	100–110 °C	> 360 °C^26^
Water solubility	43.600 mg l^−1^	33,333 mg l^−1^^26^
Molecular formula	C_16_H_18_ClN_3_S	C_32_H_22_N_6_Na_2_O_6_S_2_
Formula mass	319.85 g mol^−1^	696.7 g mol^−1^
pKa	3.14	4.5
C.l	52,015	22,120
λ_max_	664nm	496 nm

### Synthesis of absorbent

First, a single type of solid waste was examined under identical operating conditions utilizing several chemical activators. In practice, a 1/3 solid-to-activator ratio was used with a nitrogen flow rate of 100 ml min^-1^at 500 °C for one hour of operation^23^. As soon as this step was completed, the obtained carbons were sent to the surface analysis to ascertain their surface properties. The optimal combination of the solid waste and the chemical activator for the formation of activated carbon was identified based on the measured specific surface area values.

The impact of the solid-to-activator ratio on the quality of the produced activated carbons was then examined using the selected activator and a single kind of waste. This step of the investigation also employed the same operational conditions used in the previous step. This approach enabled the selection of the ideal solid-to-activator ratio for the production of activated carbons, which was then used to examine the effects of operating temperature and N_2_ flow on the properties of the resulting carbons^24^.

The operating conditions employed throughout the various phases of the activated carbon preparation process in this study are summarized in Table 3, which outlines the operational parameters applied at each stage of the activated carbon preparation.

### Characterization methods

Initially, proximate (volatile matter, ash, and moisture content) and ultimate (C, H, N, S, and O) analyses were performed on the date pits precursor and the resulting activated carbon. The ASTM D2867, ASTM D5832-98, and ASTM D2866-94 methods were used to determine the moisture, volatile matter, and ash content, respectively. The total of the ash, moisture, and volatile matter contents was subtracted from 100% to get the fixed carbon content^25^. While the oxygen concentration was determined by difference, the C, H, N, and S contents were determined using an elemental Vario Macro cube elemental analyzer.

Subsequently, the produced activated carbons were characterized using nitrogen adsorption–desorption isotherms at − 196 °C using a NOVA 3200 apparatus (USA). The samples were degassed for three hours at 200 °C under vacuum (0.13 bar). Using the BET equation, the specific surface areas (S_BET_) were computed from the adsorption branch of the isotherms.

Several analytical techniques were further used to investigate the structural, morphological, and thermal properties of the prepared activated carbon structure and the acidified catalysts that were later produced. X-ray diffraction (XRD) patterns of the two structures were recorded using Brucker AXS-D8 Advance (Germany) with nickel-filtered copper radiation (l ¼ 0.15405 nm) at 4.8 J and 40 m A with a scanning speed of 4° min^−1^ over the diffraction angle range.

In the current study, a high-resolution electron microscope (TEM), JEM 2100 (JEOL, Japan), was used, providing magnification up to 1.5 million times and electron imaging up to 0.143 nm. Energy dispersive X-ray (EDX) spectrometer (Oxford Instruments X-Max)was installed in the microscope for elemental analysis. Using an electron microscope model JM-2100-XX, JEOL-Japan, the surface and cross section morphology of the two constructions were examined. This kind of microscope can magnify objects up to 35,000 times.

The FT-IR spectra of the samples were recorded using an ATI Mattson Genesis Series spectrometer (Model 960 M009) employing the KBr disk method.

### Adsorption study

Batch adsorption studies were performed at a constant temperature of 25 °C and a fixed stirring speed of 300 rpm to eliminate external influences and ensure experimental consistency. Systematic experiments were undertaken to optimize the adsorbent dosage, initial dye concentration, and contact time required to attain adsorption equilibrium and maximum removal efficiency. 1g of MB and CR were dissolved separately in 1000 ml of distilled water to create stock solutions with a concentration of 1000 mg l^−1^. These stock solutions were successively diluted with distilled water to create the solutions of different MB and CR concentrations used in the adsorption tests. The effect of contact time was investigated within the range of 5–25 min at 5 min intervals. In addition, the effect of adsorbent dosage was investigated using different activated carbon doses of 0.10, 0.15, 0.20, and 0.25 g l^−1^.In the catalytic reduction of MB and CR, the prepared activated carbon catalytic performance was evaluated. In a standard reduction experiment, 4 ml of 40 mg l^−1^ MB (or CR) solution containing 0.02 M NaBH_4_ was stirred with 1 mg of activated carbon. Using a UV–vis spectrophotometer (JASCO, V-750 UV–visible Spectrophotometer), the absorbance spectrum of the reaction mixture was captured at predefined intervals in the 200–800 nm spectral ranges for both MB and CR.

Under the same experimental circumstances, five consecutive adsorptions–desorption cycles were used to assess the produced activated carbon’s reusability. The spent adsorbent was removed from the reaction mixture by centrifugation at 5000 rpm for five minutes following each adsorption experiment. The recovered material was then subjected to a 30-min continuous stirring treatment with 99% concentrated acetic acid to aid in the desorption of the color molecules that were retained. After continuously washing the adsorbent with deionized water until the pH reached neutral, it was oven-dried for 12 h at 70 °C before being used again. All subsequent adsorption cycles were conducted using the same initial dye concentration, adsorbent dosage, contact time, temperature 25 °C, and stirring speed (300 rpm) to ensure reproducibility and reliable comparison.

By calculating the absorbance value at 664 and 500 nm, respectively, employing a pre-constructed calibration curve for the dyes. All adsorption isotherm experiments were carried out at a constant pH of roughly 7. The quantity of dyes adsorbed per g of AC at equilibrium (q_e_, mg g^−1^) was calculated using Eq. (1).

Regarding the kinetic study, a series of experiments were carried out under conditions that were comparable to those used for the isotherm study, with the exception that the initial dye concentration was maintained at 20 mg l^−1^ throughout.

The mixtures were allowed to stir for varying amounts of time, ranging from five to twenty-five minutes. Equations (2) and (3) can be used to estimate the amount of dyes adsorbed per gram AC (q_t_, mg g^−1^) and the pollutants removal efficiency (R%), respectively. In these experiments, a dye solution containing twenty milligrams per litre was used for 20 min at a pH of 7 using 1 g l^−1^ of adsorbent.

1 $$\mathrm{R}=\frac{{\mathrm{C}}_{0}-{\mathrm{C}}_{\mathrm{e}}}{{\mathrm{C}}_{0}} \mathrm{X} 100$$

2 $${q}_{t}=\frac{{(C}_{0}-{C}_{t})V}{m}$$

3 $${q}_{e}=\frac{{(C}_{0}-{C}_{e})V}{m}$$

where R is reactivity, C_o_ is initial concentration of dye, C_e_ concentration at equilibrium, q_t_ is adsorption after time, q_e_ is adsorption at equilibrium, V volume of solution, and m is mass of used catalyst.

By conducting the adsorption process under various pH values ranging from 2 to 10 in accordance with the previously mentioned procedure, the impact of the pH of the MB and CR solutions was investigated. 0.1 M NaOH or 0.1 M HCl aqueous solutions were added to the MB and CR solutions to regulate their initial pH levels. The adsorption temperature, AC dose, initial concentration of dyes solution, and contact time were set to 25 °C, 0.2 g l^−1^, 20 mg l^−1^, 20 min, and 12.5 min for MB and CR, respectively, in these experiments. Similar to this, the effect of adsorbent dose was investigated by changing the adsorbent dose from 0.1 to 0.25 g l^−1^ while maintaining the other experimental variables, such as initial concentration of MB and CR: 20 mg l^−1^, adsorbent dose: 0.2 g l^−1^, pH: 7, temperature: 25 °C, and contact time: 20 min and 12.5 min, respectively.

### Thermodynamic study

By performing equilibrium adsorption experiments at various temperatures (25, 40, 60, and 80 °C), the impact of temperature on the adsorption process was examined. Under previously optimized adsorption conditions, a defined volume of dye solution with a known starting concentration was contacted with a predetermined amount of date pit activated carbon. A thermostatically controlled shaker was used to stir the mixtures until equilibrium was achieved. Following equilibrium, the suspensions were filtered, and spectrophotometric analysis was used to calculate the residual dye concentration (C_e_).

The mass balance equation was used to get the adsorption capacity at equilibrium (q_e_). The ratio of q_e_ to C_e_ was then used to calculate the distribution coefficient (K_d_). The linear Van’t Hoff plot of ln(K_d_) versus 1/T was used to determine the thermodynamic parameters, such as standard enthalpy change (ΔH°) and standard entropy change (ΔS°). The formula ΔG° = ΔH° − TΔS° was used to determine the standard Gibbs free energy change (ΔG°).

## Results and discussion

### Characterization of prepared materials

#### Proximate and ultimate analysis

One of the main factors used to assess the viability of the production process is the yield of AC from a specific biomass precursor, date pits, which is defined as the percentage of the residual weight of the precursor obtained at the end of the chemical activation and carbonization stage^27,28^. The yield of AC was approximately 49 wt%, meaning that date pits will produce roughly 500 kg of AC per ton of precursor. This value is consistent with those reported in the literature for AC derived from various forestry and agricultural residues^29–31^.

Table 2 presents the results of the proximate and ultimate analyses of raw date pits and AC. Date pits showed a high volatile matter content of roughly 72wt%, as shown in this table. A high content of volatile materials in waste raw materials is beneficial for the production of porous activated carbon with a large specific surface area and pore volume, which are essential requirements for an effective adsorption process. This is because the release of volatiles from the biomass precursor throughout the carbonization and activation step promotes pore formation within the resulting carbonaceous structure^32–35^.

**Table 2:** Proximate and ultimate analyses of the precursor (Date Pits) and the activated carbon developed (Date Pits AC).

Materials	Proximate analysis (wt, %)				Ultimate analysis (wt, %)			
	Moisture	Ash	Volatiles	Fixed carbon	C	H	N	O
Date Pits	8.5	3.0	72.0	16.5	46.2	4.10	1.60	48.10
Date Pits AC	6.2	3.2	15.0	75.6	75.1	1.89	0.99	22.02

Date pits were found to have a fixed carbon content of 46.2 weight percent, which is comparable to other biomass precursors commonly used to make activated carbon^32–36^. In comparison with the raw material, Table 2 showed that date pits-derived AC had much higher fixed carbon content but a lower volatile matter content. Additionally, the ash content of the date pits AC sample was estimated to be 3.2 wt%, which is marginally higher than date pits. These variations can be attributed to the release of volatile species during chemical activation, resulting in a carbonized product with higher carbon content^37,38^.

It should be noted that date pits AC ash content was significantly lower than AC maximum allowable ash content of 15 wt% according to DIN EN 12,903. High ash content not only negatively affects adsorption performance but also reduces regeneration efficiency during recycling^27,39,40^. Additionally, date pits AC carbon had a low moisture content of about 6.2 weight percent, which is within the acceptable range for activated carbon (3–6 wt%)^41^. Collectively, the proximate analysis results suggested that date pits are a perfect lignocellulosic precursor for producing high-quality activated carbon.

Table 2 also summarizes the element composition of the raw material and date pits AC. The elemental analysis showed that carbon and oxygen were the dominant elements, with only trace amounts of nitrogen and hydrogen detected. Date pits have a high carbon content of 46.2wt%, indicating their suitability as a precursor for AC production^38^. Following activation, the carbon content increased significantly from 46.2 to 75.1wt%, while the contents of the remaining elements (O, H, and N) decreased. This result can be attributed tothe evolution of the oxygen-rich volatiles from date pits during activation, leaving behind a more-stable carbon-rich structure.

#### BET analysis

**Fig. 1:**
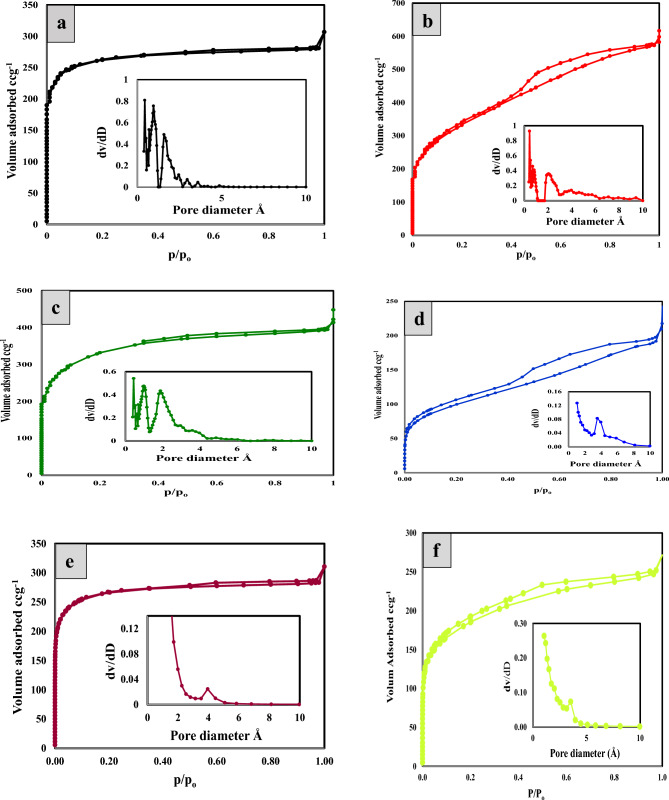

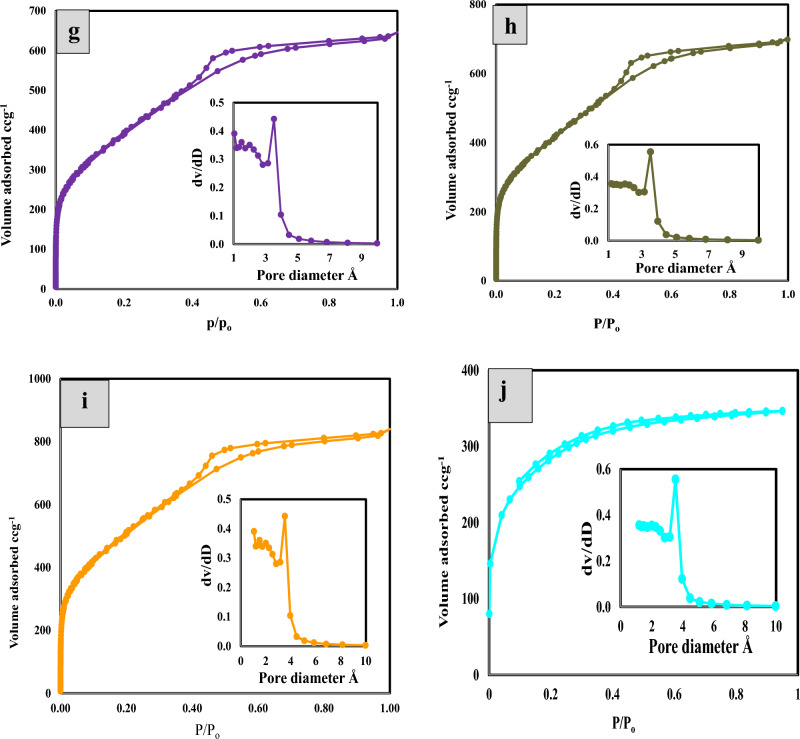
N_2_ adsorption desorption isotherms and pore size distributions of all prepared carbon materials using ZnCl_2_ and KOH as chemical activating agents at different impregnation ratios and activation temperatures, with a fixed carbonization time of 1 h.

Date pits have typically produced activated carbon with higher specific surface areas than those obtained by using the majority of other agriculture wastes, according to the data shown in Table 3 and Fig. 1a–i. This clearly relates to the type of raw material that is being used. In particular, compared to other wastes, the molecules in date pits have a less skewed building structure. Furthermore, compared to most wastes, date pits naturally contain more metals^42^. However, the type of activator has a significant impact on both the yield of the produced carbons and the values of surface area. The observable variations in the aforementioned values may be caused by the characteristics and geometry of each activator. Additionally, the layers of the biomass constituents may be displaced by the tetrahedral geometry of the chemical activators molecules, giving the resulting activated carbons a highly porous structure. The properties and geometry of each activator could be the reason for the noticeable differences in the previously mentioned values. Practically speaking, neither ZnCl_2_ nor KOH have hydrogen bonds between their molecules, which could allow their particles to be widely dispersed among the molecules of the carbon source^43^. Zinc chloride was determined to be the most effective activator for activated carbons based on the surface area values that were presented. It was then used to test other parameters that might have an impact on the properties of the generated carbons. The impact of the solid date pit-to-ZnCl_2_ ratio on the characteristics of the activated carbon was quickly investigated. The surface area values and total pore volumes of the conducted carbon show a notable increase in Table 3 up to a ratio of 1:3^43^. The solid-to-activator ratio of 1:3 was then chosen in order to investigate how the thermal conversion process operating temperature affected the characteristics of the generated carbons. First off, there was no discernible impact of the operating temperature change on the acquired carbons surface area values. Practically speaking, the surface area increased from 1550 to 1600 cm^3^ g^−1^ when the conversion temperature was lowered from 600 to 500 °C. The step of the processing temperature lower than the carbon molecules sintering temperature is responsible for the observed increase. This could actually fairly prevent the carbon molecules from coagulating during the thermal conversion process. However, the surface area value was significantly reduced to less than one-fifth of the value obtained at 500 °C when the temperature was lowered to 400 °C. As confirmed by the carbon yield value, the limited conversion rate may be due to the inadequate thermal energy supplied at that temperature; as a result, activated carbon with a very small surface area may be seen. Additionally, the study that is being presented has the potential to offer lower operating conditions in terms of temperature and time than those previously offered by the literature that is currently available. Therefore, the optimal conditions are as follows: chemical activator, zinc chloride, addition ratio of activator to waste is 3:1, operating temperature 500 °C, and burning time 1 h.

**Table 3:** Surface characteristic of the produced activated carbons at the various operating conditions.

Sample (1)	Operating condition					Results		
	Source of waste	Chemical activation	Ratio waste to activator	Temperature, ^°^C	Time	SSA, (m^2^g^−1^)	Pore diameter, (cm^3^g^−1^)	Yield, (%)
A	Date Pits	KOH	1:1	500	1h	600	0.63	62
B			1:2			700	0.73	64
C			1:3			800	0.85	65
D				400		500	0.56	65
E				600		800	0.86	45
F		ZnCl_2_	1:1	500		900	0.91	60
G			1:2			1300	1.29	62
H			1:3			1600	1.41	65
I				400		1080	1.05	68
j				600		1400	1.35	48

The adsorption–desorption isotherms of the obtained activated carbon at all operating conditions are presented in Fig. 1a–i. According to the IUPAC classification, the exhibited isotherms are for a material of type I. The shown isotherms are also suggesting that the produced activated carbon is of a highly microporous structure. Furthermore, from pore size distribution of all prepared materials, despite the presence of a mixture of micro and mesopores, show that the proportion of micropores is much larger. This indicates a high surface area of the prepared materials and confirms that the isotherm is of the type I.

#### X-ray diffraction and Raman spectroscopy of AC adsorbent

**Fig. 2:**
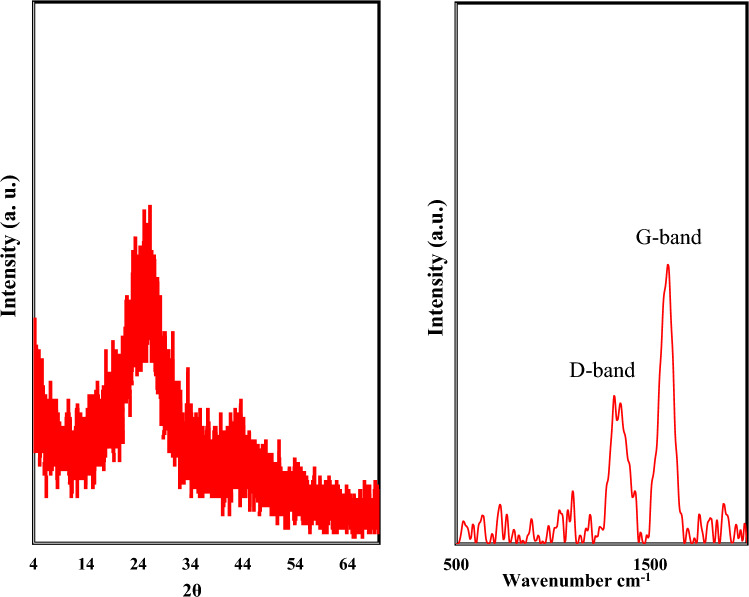
(**a**) XRD patternof the optimally prepared activated carbon. (**b**) Raman spectroscopy of the optimally prepared activated carbon.

Figure 2a shows the XRD pattern of the activated carbon structure. The XRD spectra show the presence of a broad peak between 2θ of 20.4 and 30, which is the primary indicative peak for the activated carbon matching the reflections of the (002) and (100) planes of carbon (JCPDS 75–1621), respectively^44^. The amorphous nature of the structure is generally reflected in the broad peaks. On the other hand, Fig. 2b shows the typical Raman spectrum of the obtained AC material in the 500–2000 cm^−1^ range. The well-defined D-bands (disordered or defective carbon) and G-bands (ordered or crystalline carbon) are clearly visible at wave-numbers around 1326 and 1586 cm^−1^, respectively^44^. The number of defects in the developed carbon matrix can be quantified using the integral intensities ratio of the D and G peaks (ID/IG), which for our sample equals 1.4, suggesting the presence of a high number of structural defects that are comparable to the XRD results mentioned above.

#### FT-IR spectroscopy

**Fig. 3:**
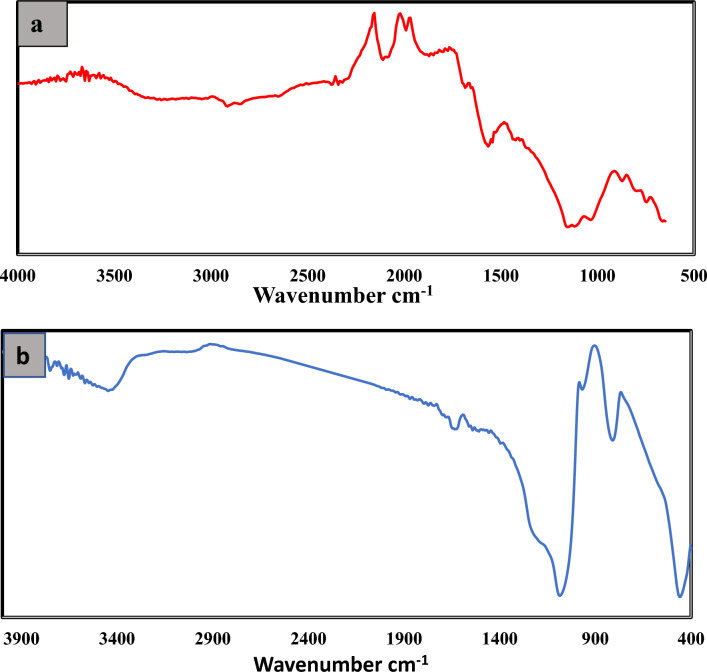
FT-IR Spectroscopy of the optimally prepared activated carbon (**a**) before the adsorption (**b**) after the adsorption.

Variations in the obtained activated carbon structure functional groups were examined using FT-IR analysis. The FT-IR technique is a helpful analytical tool that can provide precise tracking of structural changes through increases and decreases in the detected peaks intensity. Figure 3a and b show the results of the pure activated carbon FT-IR spectroscopic analysis before and after adsorption respectively. The spectrum before adsorption exhibits three main absorption bands was generally visible in the structure at approximately 3400 cm^−1^, 1100–1650 cm^−1^. At 3400 cm^−1^ in the structure, a broad band of respectable intensity is visible in the first region of absorption. The O–H stretching mode of the hydroxyl groups and any potentially present adsorbed water in the structure are responsible for this band peak. Furthermore, it should be mentioned that the bands in the 3200–3650 cm^−1^ range have also been linked to the hydrogen bonding that occurs between the OH groups^45^. The observed peak at 1640 cm^−1^ for the second major absorption is indicative of the amides that are distinguishable on the activated carbon surface. Because the biomass-based feedstock contains primary amines, the presence of such a functional group could be obtained during the activation process. This carbon fingerprint includes the peak at about 1150 cm^−1^. Additionally, C–O symmetric and asymmetric stretching vibrations of the –C–O–C– ring can be linked to this band^46^. The peak in the third region, located at 628 cm^−1^, is attributed to the bending mode of C–H. There are several bands associated with C–H bending with varying degrees of substitution in the area between 700 and 900 cm^−1^^47^.

The activated carbon bending region may suggest that the aliphatic C–H is mostly present as–CH_2_−. This can therefore make it clear that the majority of the aliphatic structures in the activated carbon are cyclic structures. After adsorption, notable changes are observed: a slight shift of the O–H and C=O peaks, a decrease in their intensity, and the appearance of new peaks around 1050–1150 cm^−1^. These changes indicate successful adsorption of the target molecules onto the functional groups of the adsorbent, suggesting interactions via hydrogen bonding and possible complex formation. The shift in peak positions and intensity reduction confirms the involvement of hydroxyl and carbonyl groups in the adsorption process.

#### HR-TEM and FESEM imaging

**Fig. 4:**
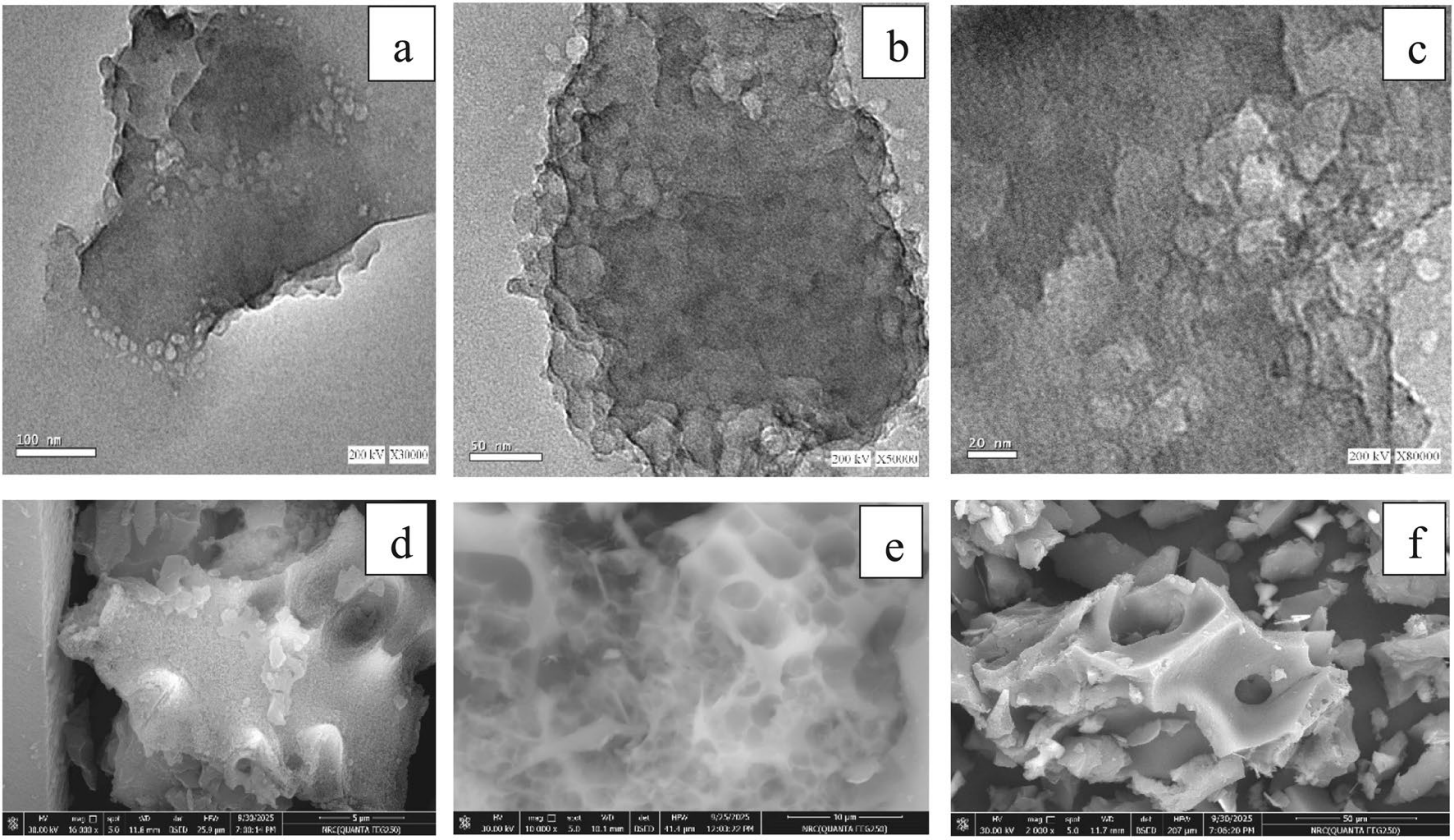
(**a**–**c**) HR-TEM images show the porous structure and layered morphology of the carbon at high magnification, indicating micro- and mesopores and (**d**, **e**, and **f**) FESEM images reveal the surface texture and particle aggregation, confirming a highly developed porous network for activated carbon prepared under optimal conditions before the adsorption.

Figure 4a–d show the prepared activated carbon surface and interior morphological characteristics. As seen in the TEM image (Fig. 4a–c), date pits AC have disordered hierarchical porosity made up of numerous micro and mesopores that appear as bright spots embedded in the amorphous matrix. Furthermore, the magnified TEM image of Date pits AC shown in Fig. 4c did not show lattice fringes. In agreement with the XRD and Raman spectroscopy findings, this result unequivocally identifies the amorphous nature of activated carbon made from date pets. The rough and uneven surface of date pits AC was revealed by the FE-SEM image Fig. 4d–f. The surface had a large number of nanoscale pores of various sizes and shapes; giving date pits AC a large surface area for the efficient removal of dyes. The release of volatile tiles as gases from the raw material (date pits) caused these void spaces to form during the chemical activation and carbonization stage^48^.

**Fig. 5:**
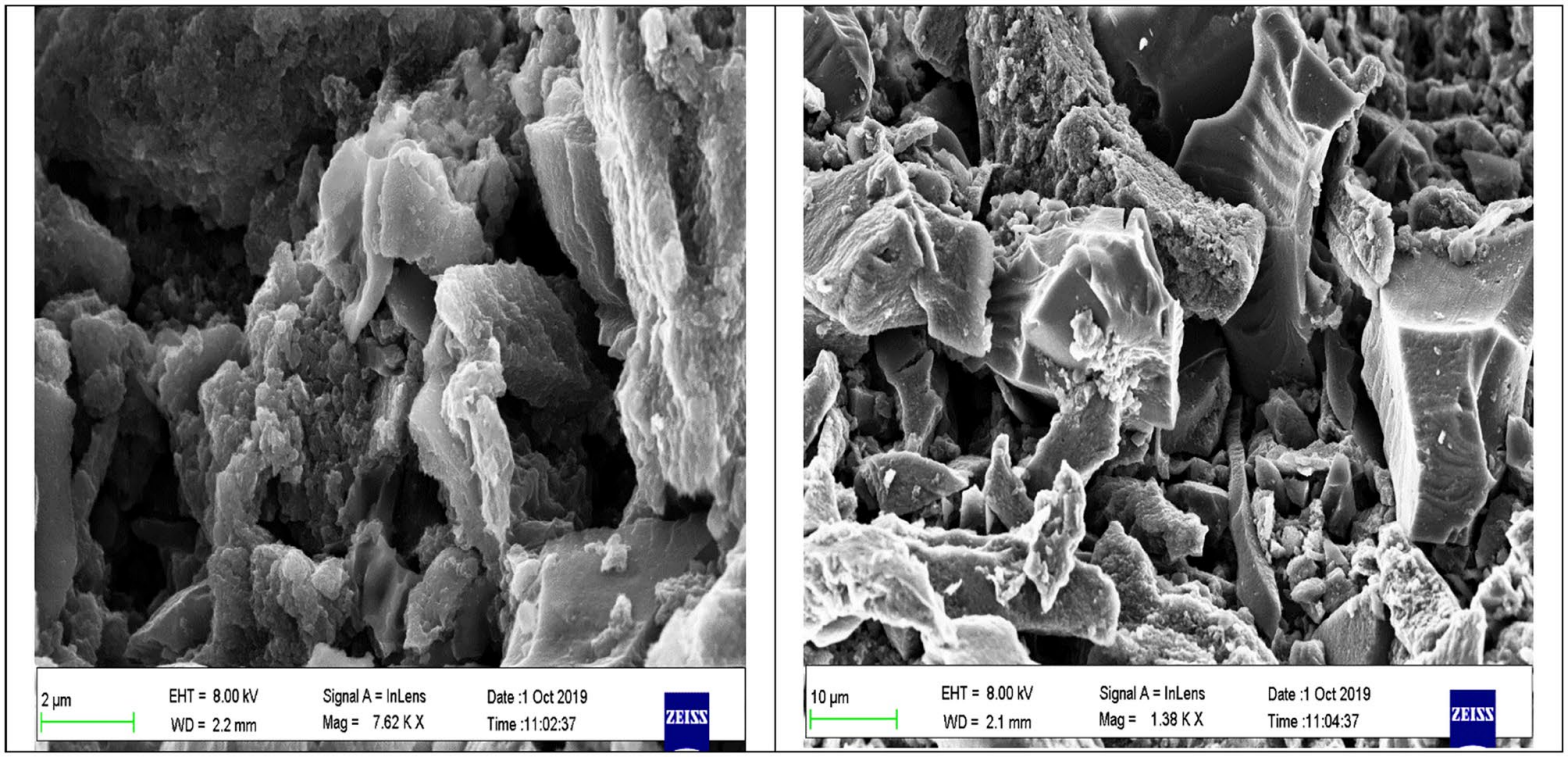
FESEM for activated carbon derived date pits after the adsorption.

After adsorption, the surface of the activated carbon appears partially covered by dye molecules, with reduced visible pore openings compared to the fresh sample. This observation suggests pore filling and surface coverage effects, confirming successful dye uptake as shown in Fig. 5.

EDX analysis was performed on the prepared adsorbent prior to dye adsorption to evaluate its elemental composition (atomic %). The results revealed that the adsorbent primarily consists of carbon (C K) 79.44%, oxygen (O K) 17.05%, sodium (Na K) 0,60% which indicates the presence of a carbonaceous matrix with oxygen-containing functional groups capable of interacting with dye molecules.

### Adsorption of MB and CR by activated carbon

#### Effect of adsorbent dose

**Fig. 6:**
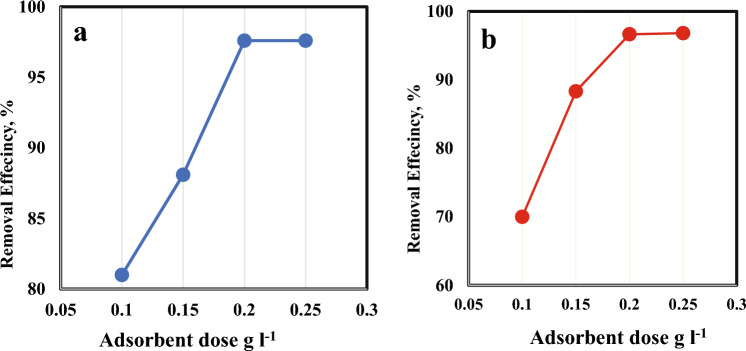
Effect of Adsorbent dose for (**a**) methylene blue and (**b**) Congo red.

Figure 6a and b show the results of a thorough investigation into the impact of adsorbent dose on the removal of Methylene Blue and Congo Red by AC. It was found that when the adsorbent dose was increased from 0.1 to 0.25 g l^−1^, the methylene blue sorption rate increased from 81% to 97.6%. The anticipated increase in the contact area available for MB sorption and the total number of exposed binding sites were essentially linked to the beneficial effect of the AC dose^49^. Therefore, a higher MB removal could be achieved by AC capturing more MB. Nevertheless, the sorption rate hardly changes when the adsorbent dose is increased above 0.2 g l^−1^. Considering these findings, AC dose of 0.2 g l^−1^ was selected for the continuation of the study.

However, by changing the dosage of AC from 0.1 to 0.25 g l^−1^ at a fixed initial concentration of 20 mg l^−1^, the impact of adsorbent dosage on CR removal from aqueous solutions was investigated. Figure 6b displays the corresponding results. It demonstrated that raising the dosage of the adsorbent could improve the solution color removal effectiveness. The removal of CR increased with increasing adsorbent dosage because the adsorbent surface area increased, facilitating easier penetration of the adsorbate to the adsorption sites^50^.

In addition to removal efficiency, the effect of adsorbent dose on adsorption capacity (qe) was also evaluated. Availability of active adsorption sites, the adsorption capacity (qe) showed a decreasing trend. This can be attributed to the partial utilization of adsorption sites at higher dosages, particle aggregation, and a decrease in the effective concentration gradient. Similar behaviour has been widely reported in adsorption studies.

#### Effect of adsorbent dose on adsorption capacity (q_e_)

**Fig. 7:**
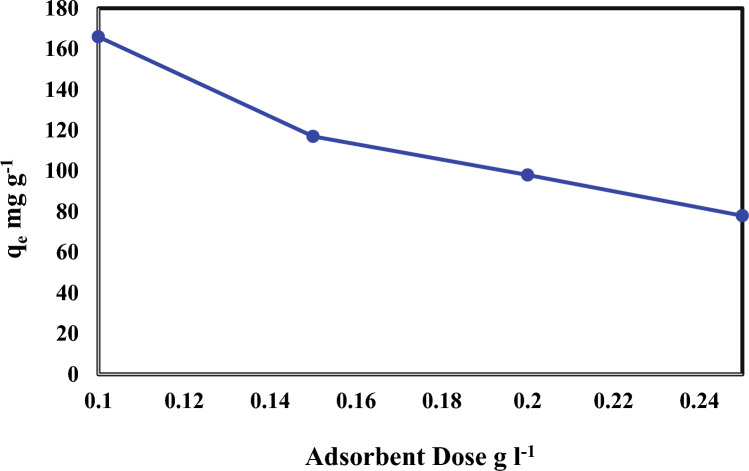
Effect of adsorbent dosage on the adsorption capacity (q_e_) of methylene blue onto date-pits activated carbon.

The impact of adsorbent dosage on the methylene blue adsorption capacity (q_e_) onto date-pit activated carbon is shown in Fig. 7. From 166 mg g^−1^ at an adsorbent dosage of 0.1 g l^−1^ to 78 mg g^−1^ at 0.25 g l^−1^, the adsorption capacity gradually dropped. This behaviour, which is frequently seen in adsorption systems, can be explained by the fact that as adsorbent dosage increases, more adsorption sites become available. As a result, active sites are not fully utilized, and less adsorbate is adsorbed per unit mass of adsorbent. Additionally, the effective surface area available for adsorption may be decreased by particle aggregation at higher adsorbent dosages.The distribution of dye molecules over a greater mass of adsorbent caused the adsorption capacity to decrease even though the removal efficiency increased with increasing adsorbent dosage.

Although the removal efficiency increased with increasing adsorbent dose, the adsorption capacity (q_e_) showed the opposite trend. This is because the available adsorption sites increased more rapidly than the amount of dye present in the solution, leading to a lower dye uptake per unit mass of adsorbent.

**Fig. 8:**
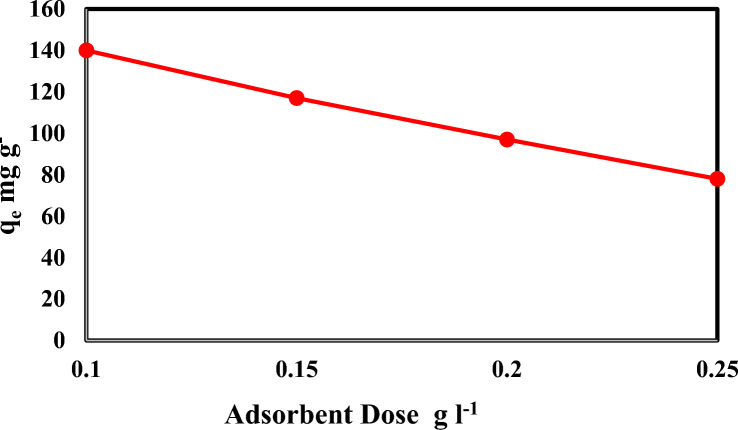
Effect of adsorbent dosage on the adsorption capacity (q_e_) of Congo red onto date-pits activated carbon.

The impact of adsorbent dosage on Congo red’s adsorption capacity (q_e_) onto date pit activated carbon is shown in Fig. 8. At an adsorbent dosage of 0.1 g l^−1^, the adsorption capacity was 140 mg g^−1^; at 0.25 g l^−1^, it was 78 mg g^−1^.

This tendency is frequently seen in adsorption systems and is explained by the fact that as adsorbent dosage increases, the number of available adsorption sites increases. As a result, active sites are not fully utilized, and the amount of adsorbate adsorbed per unit mass of adsorbent decreases. Furthermore, the effective surface area available for adsorption may be decreased by particle aggregation at higher dosages. The distribution of dye molecules over a greater mass of adsorbent caused the adsorption capacity to decrease even though the removal efficiency increased with increasing adsorbent dosage.

#### Effect of initial pH

**Fig. 9:**
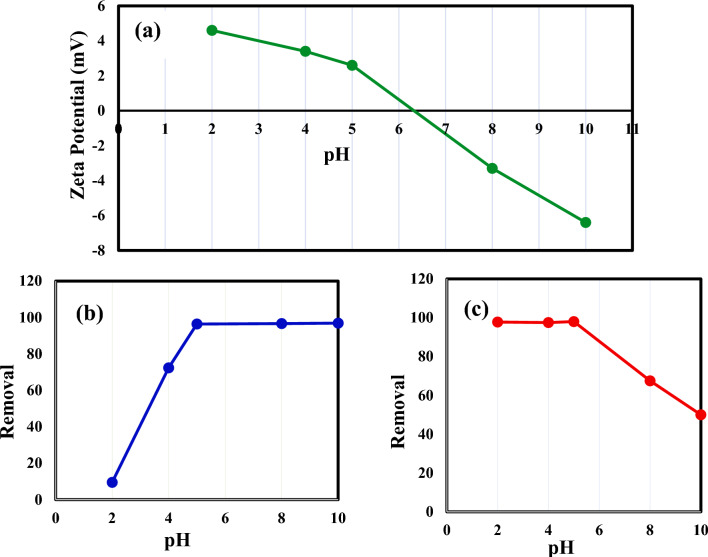
Effect of (**a**) The pHpzc values of date pits AC, (**b**) pH on the uptake of MB onto date pits AC, and (**c**) pH on the uptake of CR onto date pits AC.

The pH of the solution is one of the key factors that significantly affect dye adsorption because of various mechanisms like protonation processes between dyes and the adsorbent surface. The adsorbent surface charge and the adsorbate ionization are influenced by the pH of the solution. However, the adsorption performance in aqueous solutions is also influenced by hydrophobic interaction, hydrogen bonds, π-π interaction, and n-π interaction. Figure 9b illustrates the effects of pH for CR dyes. At pH 5, the removal rate of CR was highest, with nearly 100% uptake. At pH 5, date pits AC effectively removes CR, which is negatively charged. The negatively charged CR dye and an aqueous solution with a low pH (high H^+^ charge) are attracted to each other, increasing the adsorption efficiency^51,52^. Numerous studies have also reported similar outcomes for the adsorption of CR^53–56^. Barkauskas et al.^57^ and Debnath et al.^58^ state that CR employs two mechanisms in the adsorption process: π-π interaction between the dye’s aromatic rings and the basal planes of the date pits AC. Hydrogen bonds between oxygen and the hydroxyl groups of the adsorbent, oxygen, and the amino groups of CR are also present. The highest adsorption uptake of CR occurs when the adsorbent surface charge is at its lowest point (pH 5), which permits the ionization and protonation of its functional groups to occur at their maximum rate. However, as anticipated given its cationic characteristics, MB dye demonstrated outstanding adsorption in a basic medium, as seen in Fig. 9c. There was less competition between positive ions and dye molecules as the pH rose because the H^+^ charges in the solution dropped. Due to the date pits AC negative charge at high pH, there is a greater electrostatic force of gravity between the adsorbent surface and the cationic dye molecule, which increases adsorption efficiency. Figure 9a displays the pHpzc values for date pits AC 5.6. This showed that cationic species (MB) tend to absorb on the surface of negatively charged adsorbents (pH > pHpzc) likely involved electrostatic interactions, whereas positively charged adsorbent surfaces tend to absorb anionic species or negatively charged dye (in this case, CR) when pH < pHpzc^51^. Furthermore, the variation of adsorption capacity (q_e_) with pH was analysed to better understand the adsorption behaviour. The solution pH significantly influenced the adsorption capacity by altering the surface charge of the adsorbent and the ionization degree of the dye molecules. At optimal pH values, strong electrostatic attraction enhanced the adsorption capacity, while at unfavourable pH conditions, electrostatic repulsion and competition with hydrogen or hydroxide ions resulted in lower q_e_ values.

#### Effect of pH solution on adsorption capacity (q_e_)

**Fig. 10:**
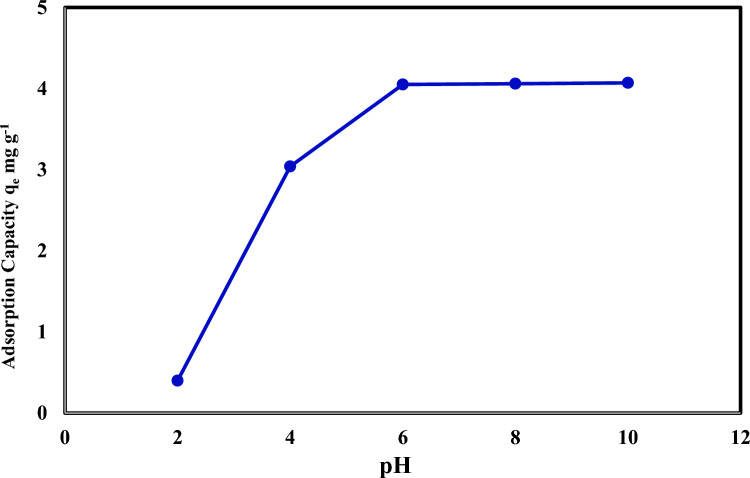
Effect of solution pH on the adsorption capacity (q_e_) of methylene blue onto date-pits activated carbon.

The effect of solution pH on the adsorption capacity (q_e_) of methylene blue onto date-pit activated carbon is shown in Fig. 10. The adsorption capacity increased significantly from 0.40 mg g^−1^ at pH 2 to 4.05 mg g^−1^ at pH 6, which indicated that the solution pH has a significant effect on adsorption process. The high concentration of H^+^ ions in acidic medium compete with methylene blue molecules for the available adsorption sites, which causes the decrease in the adsorption capacity. The competition from H^+^ ions gradually reduced, and more dye molecules could interact with the surface of adsorbent with increasing pH.

Moreover, at the solution pH value higher than the pHpzc value (5.6), the surface of the activated carbon was negatively charged and increased the electrostatic attraction to the positively charged methylene blue molecules. Thus, the adsorption capacity was significantly increased and reached its plateau at pH ≥ 6 indicating that the adsorption process was likely to reach equilibrium and the readily available active sites were almost saturated. These results are in agreement with the results of removal efficiency and confirm that slightly alkaline conditions are favorable for the methylene blue adsorption on the prepared activated carbon.

**Fig. 11:**
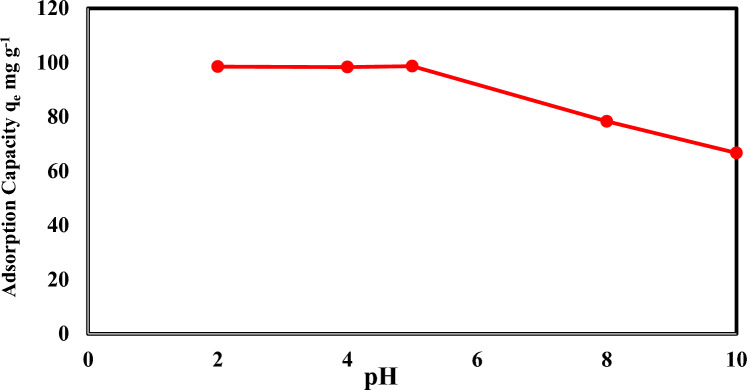
Effect of solution pH on the adsorption capacity (qe) of Congo red onto date-pits activated carbon.

Figure 11, on the other hand, illustrates the effect of solution pH on the adsorption capacity (q_e_) of Congo red on the activated carbon. Adsorption capacity was in the range of 98.5–98.7 mg g^−1^ at acidic pH (2–5). However, a significant decrease in the adsorption capacity was noticed at alkaline pH values which decreased to 78.3 and 66.7 mg g^−1^ at pH 8 and 10, respectively. The observed behavior can be explained due to the anionic nature of the congo red molecules. At pH values lower than the pHpzc (5.6), the surface of activated carbon is positively charged, which results in a strong electrostatic attraction with the negatively charged sulfonate groups of Congo red. On the other hand, the surface of the adsorbent is negatively charged at pH values higher than pHpzc, which leads to electrostatic repulsion and a decrease in the adsorption capacity.

The obtained results show the importance of the electrostatic interactions in the adsorption mechanism and confirm the affinity of the Congo red to acidic conditions.

Finally, the ionic properties of methylene blue and Congo red are different, and their adsorption behaviours at different pH values were compared, showing opposite trends. Congo red had low adsorption capacity at alkaline pH values and high adsorption capacity under acidic conditions, while methylene blue’s adsorption capacity increased with increase in pH. This different behaviour was directly related to the pHpzc value of 5.6 of the activated carbon prepared. The adsorbent surface is positively charged under the pHpzc and benefits the adsorption of anionic Congo red molecules; while the surface becomes negatively charged above the pHpzc and benefits the adsorption of cationic methylene blue molecules.

#### Effect of contact time

**Fig. 12:**
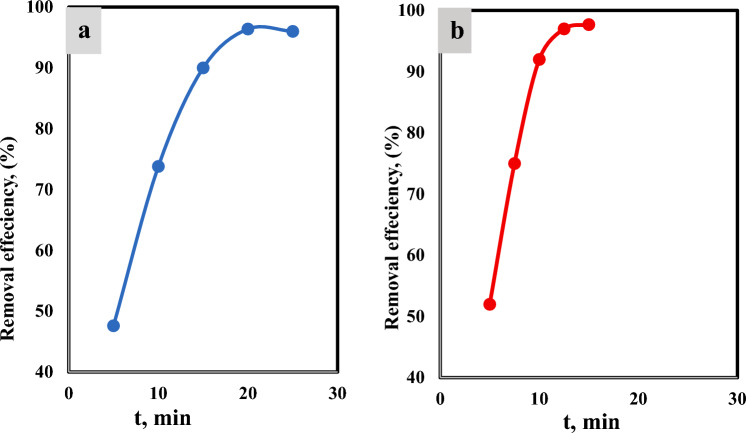
Effect of contact time on removal efficiency for (**a**) methylene blue (**b**) Congo red.

For both dyes, the removal efficiency significantly increased with increasing contact time under the testing circumstances (25 °C, pH 5.6, and constant initial dye concentration) (Fig. 12). The clearance effectiveness for MB (Fig. 12a) rose from roughly 48% after 5 min to roughly 96% after 20 min; no further increase was seen after that, suggesting that adsorption equilibrium had been reached. Comparably, for CR (Fig. 12b), the clearance effectiveness rose from roughly 52% at 5 min to almost 98% in 12–15 min, indicating a quicker rate of adsorption than for MB. The strong concentration gradient between the solution and adsorbent surface, which encourages mass transfer, and the large number of empty active sites are responsible for the quick adsorption seen in the first step. The available adsorption sites gradually filled up as contact time increased, slowing the rate of adsorption until equilibrium was attained. The produced activated carbon’s remarkable affinity for MB and CR molecules is demonstrated by the high removal efficiencies attained for both dyes.

#### MB and CR adsorption isotherms

**Fig. 13:**
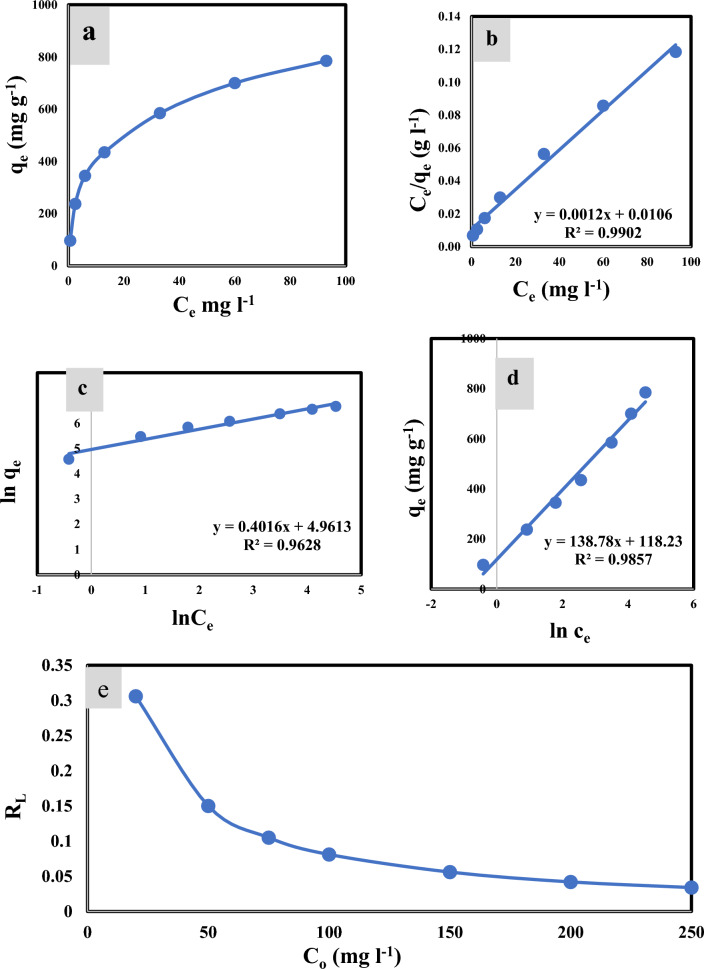
(**a**) Adsorption isotherm of MB over date pits AC, (**b**) Langmuir, (**c**) Freundlich, and (**d**) Temkin isotherm plots for MB adsorption over AC at 25 °C, and (**e**) variation of the Langmuir separation factor (R_L_) as a function of initial AC concentration.

Studying the equilibrium adsorption isotherm is essential to comprehending the mechanism that drives the adsorption process. Figure 13a shows the adsorption isotherms that were produced by graphing the equilibrium adsorption capacity (q_e_) against the MB concentration (C_e_). The findings showed that at lower MB concentrations, the amounts of MB adsorbed onto the material increased quickly, improved more slowly, and ultimately plateaued. Three models, the Langmuir (Eq. 4), Freundlich (Eq. 5), and Temkin (Eq. 6) isotherms, were employed in this study to examine the experimental adsorption data in order to gain a better understanding of the interactions between MB species and the date pit AC surface.

4 $$\frac{{C}_{e}}{{q}_{e}}=\frac{1}{{q}_{max}{K}_{L}}+\frac{{C}_{e}}{{q}_{max}}$$

5 $$ln{q}_{e }=ln{K}_{F }+ \frac{1}{n}ln{C}_{e}$$

6 $${q}_{e }=BlnA+Bln{C}_{e}$$

where q_max_ (mg g^−1^) is the maximum adsorption capacity; K_L_ (l mg^−1^) stands for constant of Langmuir equilibrium related to the energy of adsorption; K_F_ (l g^−1^) denotes the Freundlich constant representing the maximum adsorption capacity; n constants related to the favourability of the adsorption pro cess, A (mg l^−1^) is the equilibrium constant related to the binding energy, and B is the Temkin constant related to the adsorption heat.

The information in Table 4 shows that the equilibrium data can be more accurately represented by the Langmuir model than by the Freundlich and Temkin models. This is predicated on the adsorption of dye species as a monolayer across an even adsorbent surface and the nearly identical activation energies of the adsorption of the MB and CR molecules^51,59^. There was no lateral interaction between the adsorbed species for the Langmuir adsorption isotherm^60^. The experiments did not achieve the theoretical maximum adsorption capacity of MB and CR onto date pit adsorbent, according to the positive value of the Langmuir constant, b^61^.

**Table 4:** Adsorption isotherm parameters.

Isotherm		MB	CR
	Parameters	Results	Results
Langmuir	R^2^	0.990	0.984
	K_L_ (l mg^−1^)	0.113	0.135
	q_max_ (mg g^−1^)	833.30	740.00
Freundlich	R^2^	0.960	0.983
	K_F_ ((mg g^−1^) (l mg^−1^)1/n)	49.00	94.70
	N	1.80	2.38
Temkin	R^2^	0.980	0.920
	A (mg l^−1^)	10^5^*2.2	136.02
	B	6.44	1.12

Additionally, the value of a dimensionless constant related to the Langmuir model, the separation factor, or R_L_, can provide information about how favourable the current sorption process is^62^.

**Fig. 14:**
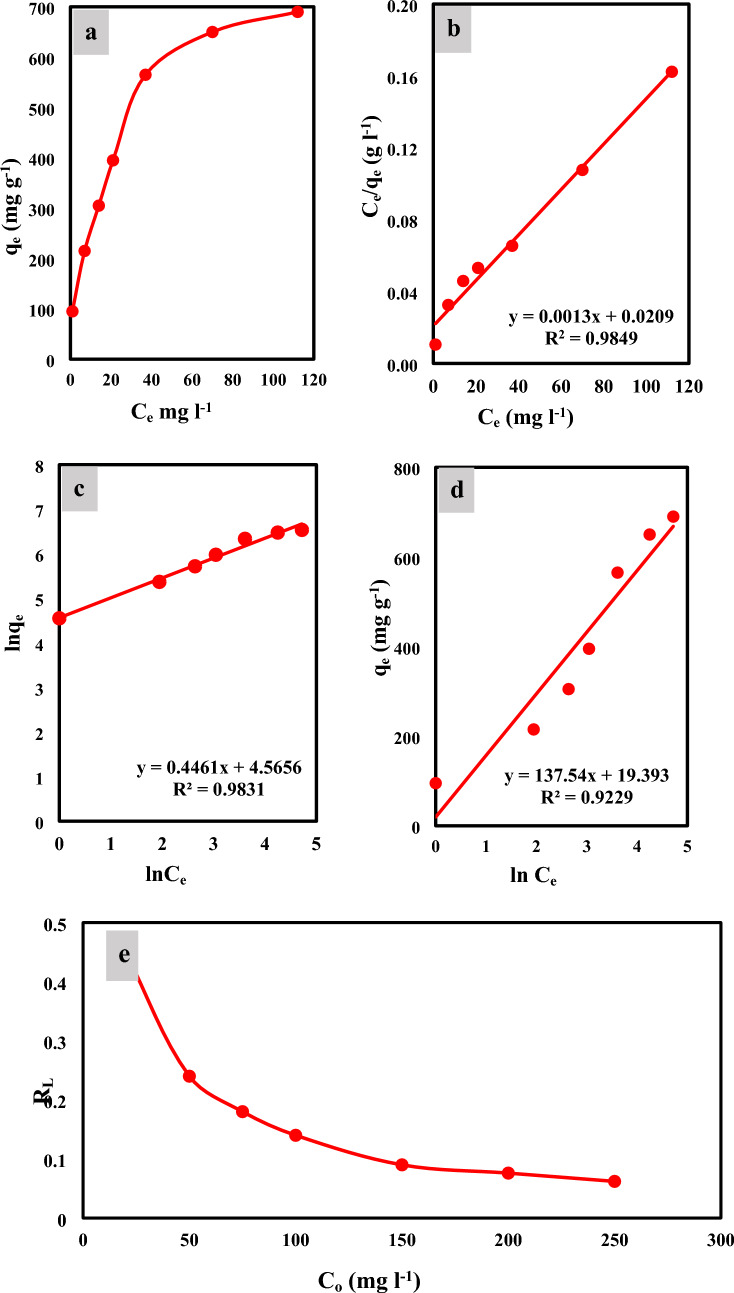
(**a**) Adsorption isotherm of CR over date pits AC, (**b**) Langmuir, (**c**) Freundlich, and (**d**) Temkin isotherm plots for CR adsorption over AC at 25 °C, and (**e**) variation of the Langmuir separation factor (R_L_) as a function of initial AC concentration. Figures 8b, c, and d display the fitting results, and Table 4 outlines the pertinent parameters.

Figures 13e and 14e show the R_L_ values at various MB and CR initial concentrations that were calculated using Eq. 7. Assuming that the sorption of both MB and CR species onto date pits AC is a favourable sorption process for all of the studied concentrations, the RL value in the current study was found to range from 0.062 to 0.45^63^.

7 $${R}_{l = }1/(1+ {k}_{l}+ {C}_{o})$$

#### MB and CR adsorption Kinetics

**Fig. 15:**
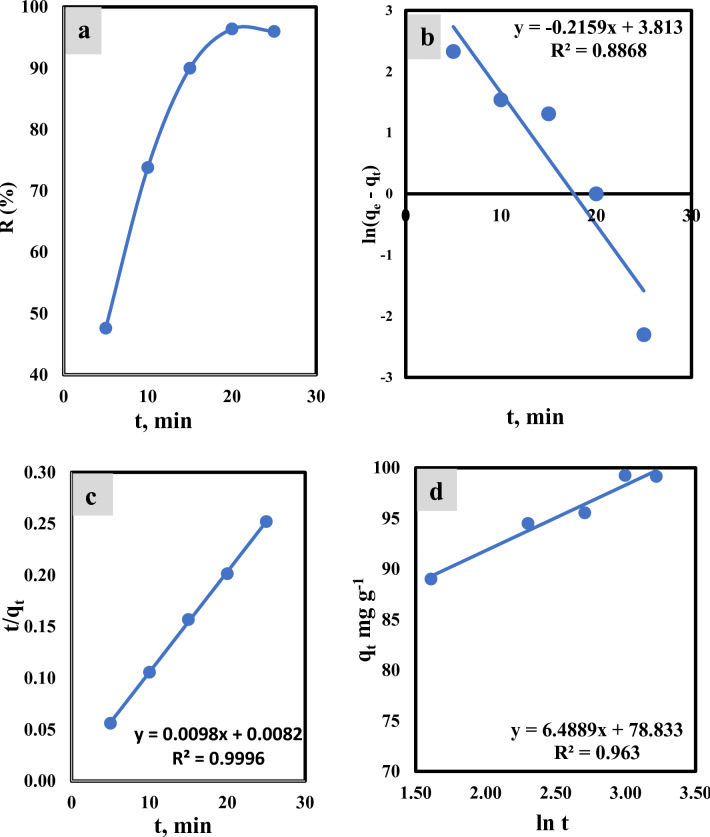
(**a**) Effect of adsorption time on the uptake of MB onto date pits AC. Conditions: initial MB concentration, 20 mg l^−1^; adsorbent dosage, 0.2 g l^−1^; pH, 7; 298 K. (**b**) pseudo-first-order, (**c**) pseudo-second-order, and (**d**) Elovich linear plots for the removal of MB onto date pits AC.

**Fig. 16:**
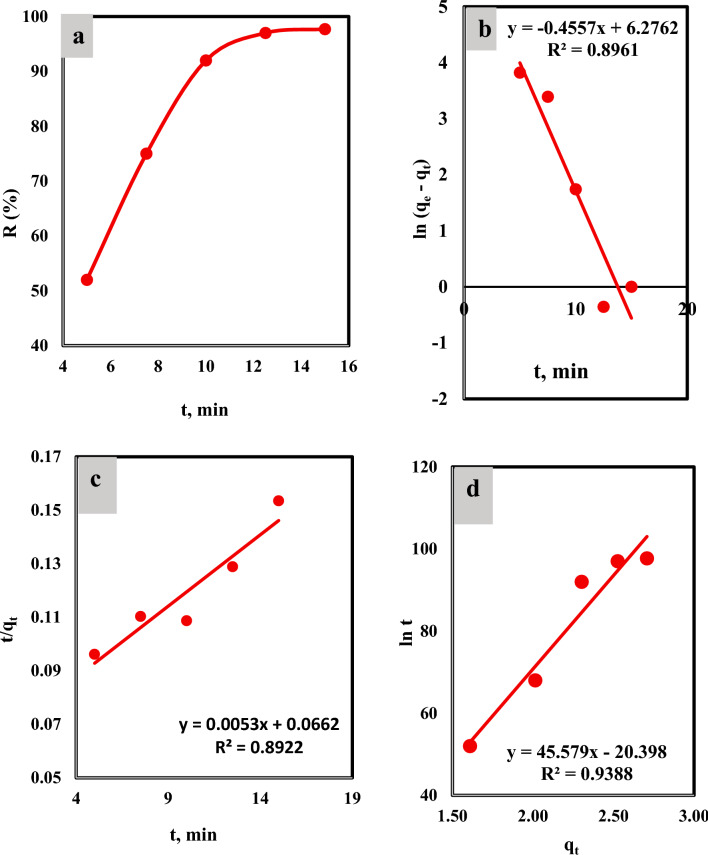
(**a**) Effect of adsorption time on the uptake of CR onto date pits AC. Conditions: initial CR concentration, 20 mg l^−1^; adsorbent dosage, 0.2 g l^−1^; pH, 7; 298 K. (**b**) pseudo-first-order, (**c**) pseudo-second-order, and (d) Elovich linear plots for the removal of CR onto date pits AC.

Figures 15a and 16a shows how the adsorption of both MB and CR on date pits AC varies as a function of contact time. The figures show that the rate of dye removal was quick, and adsorption equilibrium could be reached after 20 and 12.5 min respectively. It is evident that the adsorption rate was rapid before progressively slowing down over time and eventually reaching equilibrium and a plateau. This behaviour can be explained by the fact that dye molecules could readily enter the binding centres and adsorb quickly because the occupation of the surface-active binding sites was low at the beginning of the process^47^. Dye molecules on the date pits AC surface would electrostatically repel those that are free in the solution as the adsorption process progressed and the number of free, unoccupied active binding sites decreased.

The pseudo-first-order, pseudo-second-order, and Elovich models were fitted to the kinetic data of dyes removal by date pits AC in this study. The linear formula for these models is expressed in Eqs. 8, 9, and 10.

For both MB and CR dyes, the fitting adsorption data to the three kinetic models are shown in Figs. 15b to d and 16b to d, respectively.

From the data shown in Figures, the kinetic parameter values and the determination coefficients, R^2^, were calculated and assembled in Table 5. The pseudo-second-order kinetic model appears to better fit the actual data for both MB adsorption based on the values of R^2^, and the computed q_e_ value (q_e_, cal) from this kinetic model was consistent with the experimental q_e_ value (q_e_, exp).

**Table 5:** Fitted parameters of MB and CR adsorption kinetics on date pits AC.

Model	Parameters	MB	CR
		Results	Results
Pseudo-first-order model	K_1_ (min^−1^)	0.215	0.4557
	q_e, theo_ (mg g^−1^)	45.3	533
	R^2^	0.886	0.896
Pseudo-second-order model	K_F_ ((mg g^−1^) (l mg^−1^)1/n)	0.0117	4.2*10^–4^
	q_e,theo_ (mg g^−1^)	102	189
	R^2^	0.999	0.892
Elovich kinetic model	A	10^6^*1.3	29.2
	B	0.155	0.0219
	R^2^	0.963	0.938

Based on both statistical fitting and physical meaning, the pseudo-second-order model more accurately described the adsorption kinetics, even though the Elovich model for CR dyes displayed a higher correlation coefficient. These results suggest that chemisorption could contribute significantly to the dye adsorption process in this investigation^47,64^.

8 $$\mathrm{l}\mathrm{n}({{q}_{e } - q}_{t })={ln q}_{e }- {K}_{1 }t$$

9 $$\frac{t}{{q}_{t}}=\frac{1}{{k}_{2}({q}_{e}{)}^{2}}+\frac{t}{{q}_{e}}$$

10 $${q}_{t}=\frac{1}{\beta }\mathit{ln}\alpha \beta +\frac{1}{\beta }\mathit{ln}t$$

where k_1_ (min^−1^) and k_2_ (g mg^-1^min^−1^) are the equilibrium rate constants of pseudo-first- and pseudo-second-order rate equation, respectively, β (g mg^−1^) is the desorption constant, and α (mg g^-1^min^−1^) is the initial adsorption rate.

#### Thermodynamic study of MB

The thermodynamic behavior of the adsorption process was investigated at different temperatures (25, 40, 60, and 80 °C). The distribution coefficient (K_d_) was calculated according to:

11 $${\mathrm{K}}_{{\mathrm{d}}} = {\mathrm{q}}_{{\mathrm{e}}} /{\mathrm{C}}_{{\mathrm{e}}}$$

where q_e_ (mg g^−1^) is the adsorption capacity at equilibrium and C_e_ (mg l^−1^) is the equilibrium concentration of the dye in solution.

The standard thermodynamic parameters were determined using the Van’t Hoff equation:

12 $${\text{ln K}}_{{\mathrm{d}}} = \left( {\Delta {\mathrm{S}}^\circ /{\mathrm{R}}} \right) - \left( {\Delta {\mathrm{H}}^\circ /{\mathrm{RT}}} \right)$$

where ΔH° (kJ mol^−1^) is the standard enthalpy change, ΔS° (J mol^−1^ K^−1^) is the standard entropy change, R is the universal gas constant (8.314 J mol^−1^ K^−1^), and T is the absolute temperature (K). The Gibbs free energy change was calculated using:

13 $$\Delta {\mathrm{G}}^\circ = \Delta {\mathrm{H}}^\circ - {\mathrm{T}}\Delta {\mathrm{S}}^\circ$$

The thermodynamic parameters obtained from the Van’t Hoff plot are presented in Table 6.

**Table 6:** Thermodynamic parameters for the adsorption process.

Parameter	Value
ΔH° (kJ mol^−1^)	− 31.1
ΔS° (J mol^−1^ K^−1^)	− 62.8

**Fig. 17:**
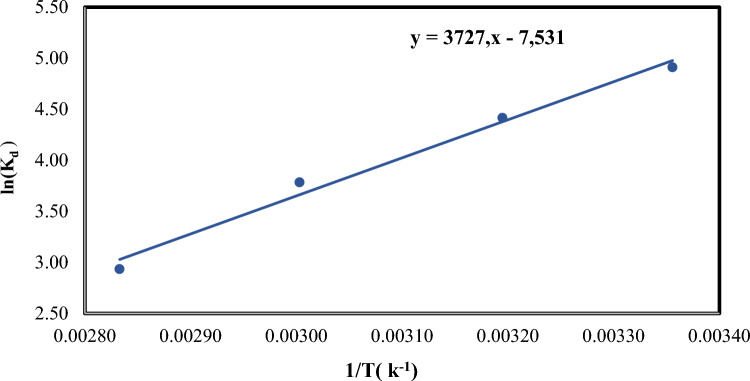
Van’t Hoff plot for the estimation of thermodynamic parameters.

The negative values of ΔG° at all investigated temperatures indicate that the adsorption process occurred spontaneously (Table 7). The negative enthalpy change (ΔH° = − 31.1 kJ mol^−1^) confirms the exothermic nature of the adsorption process. Moreover, the negative value of ΔS° suggests a decrease in randomness at the solid–liquid interface during adsorption. The decrease in K_d_ values with increasing temperature indicates that adsorption becomes less favourable at elevated temperatures, which is consistent with the exothermic behaviour of the system. Figure 17. shows the Vant Hoff plot obtained from the relationship between ln(Kd) and 1/T, from which the thermodynamic parameters were estimated.

**Table 7:** Gibbs free energy values at different temperatures.

Temperature (K)	ΔG° (kJ mol^−1^)
298	− 12.34
313	− 11.40
333	− 10.19
353	− 8.93

#### Thermodynamic study of CR

In the temperature range of 298–353 K, the impact of temperature on Congo Red adsorption was examined. The thermodynamic behavior of the adsorption system was assessed using the distribution coefficient (K_d_), which was computed from the equilibrium adsorption data. At 298, 313, 333, and 353 K, the obtained K_d_ values were 195.00, 328.33, 272.78, and 212.39. Adsorption affinity was found to slightly increase with temperature up to 313 K, after which it gradually decreased at higher temperatures. This behavior points to the existence of an ideal adsorption temperature of about 313 K. Over the examined temperature range, the adsorption capacity showed only slight variations, suggesting that temperature had little effect on the adsorption process. The combination of increased dye mobility at moderate temperatures and the partial weakening of adsorbent–adsorbate interactions at higher temperatures may be responsible for the observed trend. Overall, the thermodynamic results show that Congo Red adsorption remained very effective across the temperature range under investigation, with temperature having very little effect on the adsorption performance.

#### Comparative adsorption behaviour of MB and CR

The observed difference in adsorption behavior between methylene blue (MB) and Congo red (CR) can be attributed to their molecular structures, charge characteristics, and diffusion properties. MB is a smaller cationic dye with a relatively planar structure, which enhances electrostatic attraction toward the negatively charged adsorbent surface and facilitates diffusion into micropores. Additionally, π–π interactions between the aromatic rings of MB and the carbonaceous surface may have contributed to the adsorption process. In contrast, CR is a larger anionic dye with a more complex molecular structure and higher steric hindrance, which limits its accessibility to internal pores and results in greater diffusion resistance. Consequently, CR adsorption predominantly occurs on the external surface and may rely more significantly on weaker interactions such as hydrogen bonding and van der Waals forces. These mechanistic differences explain the lower adsorption capacity and slower adsorption kinetics observed for CR compared to MB.

#### Reusability

**Fig. 18:**
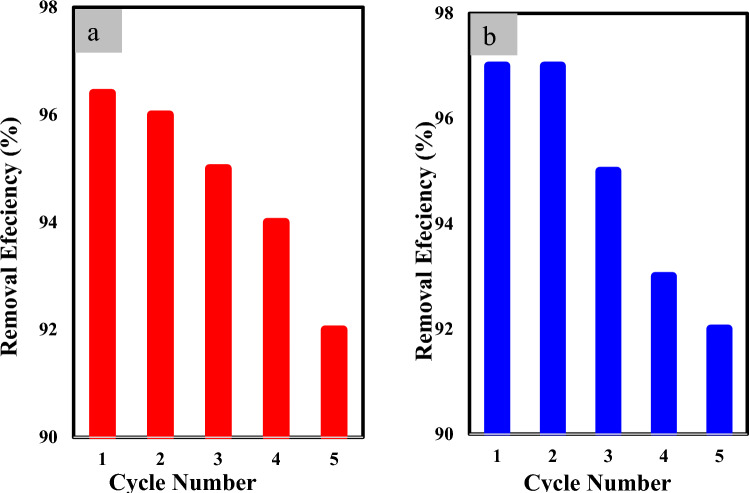
Reusability of date palm AC of (**a**) MB and (**b**) CR.

Reusability is another important factor that needs to be considered when assessing a material’s adsorption performance from the perspective of practical application. To assess the long-term operational stability of the prepared date pits activated carbon, five consecutive adsorption–desorption cycles were conducted under identical experimental conditions; the outcomes of these experiments are displayed in Fig. 18. The adsorption efficiencies of MB and CR on date pits AC only decreased by 5% after five consecutive runs, from 95.6% (after the first run) to 92% (after the fifth run), demonstrating the strong operational stability and reusability of date pits AC during the adsorption of MB and CR. This limited decline in adsorption efficiency indicates that the structural framework and surface functional groups of the activated carbon remained largely preserved during repeated regeneration and reuse. The high retention of adsorption performance suggests that the porous structure was not significantly collapsed and that the majority of active adsorption sites remained accessible after successive cycles. Overall, the results confirm that the prepared activated carbon exhibits excellent reusability and operational stability, highlighting its strong potential for sustainable and cost-effective dye removal in practical wastewater treatment systems.

#### Comparison study

Interms of the attained maximum uptake capacities for MB and CR, the date pits AC adsorbent performance was contrasted with that of other carbon adsorbents made from different agricultural and forestry residues documented in the literature (Table 8). Compared to the adsorbents listed in Table 8, the maximum uptake capacity of MB on date pits AC was 833 mg g^−1^, and CR was 740 mg g^−1^, which were higher than many previously reported adsorbents. Because of its simple synthesis process, exceptional saturation adsorption capacity, quick adsorption kinetics, and outstanding reusability, the activated carbon produced from date pits using ZnCl_2_ activation was therefore appealing for the removal of both MB and CR from water media.

**Table 8:** Comparison of maximum uptake capacities of various adsorbents for dyes.

Dyes	Adsorbent	q_max_	References
MB	AC from Corn stigmata	106.30	^65^
	AC from Rice straw	20.62	^66^
	AC from Ephedra strobilacea	31.152	^67^
	AC from Luffa cylindrica	49.46	^68^
	AC from Canola residues	32.21	^69^
	AC from Coconut husks	418.15	^70^
	AC from date pits	833.3	Current work
CR	AC from Spear grass leaves	318	^71^
	AC from coffee waste	90.90	^72^
	AC from Peanut shells	150	^73^
	AC from Grape wastes	455	^55^
	AC from date pits	740	Current work

## Conclusion

An efficient environmentally friendly biosorbent for the removal of cationic (MB) and anionic (CR) dyes from aqueous solutions is activated carbon, which is obtained from agricultural waste biomass and the production of date pits. The set of experiments determined the ideal biosorption conditions for methylene blue dye: initial pH > 5.0; contact time of 20 min; biosorbent dosage of 0.2 g l^−1^; initial concentration range of 25 to 250 ppm; and temperature of 298 K. CR dye, on the other hand, has an initial pH of less than 5.0, a contact time of 12.5 min, a biosorbent dosage of 0.2 g l^−1^, an initial concentration range of 25 to 250 ppm, and a temperature of 298 K. The primary benefit of the pre-treated biosorbent is that the anionic dye’s biosorption is largely unaffected by pH. Because it is expensive and challenging to lower the wastewater pH to an acidic level, this offers a potential practical application. Langmuir models fit the equilibrium data for MB and CR absorption quite well. The absorption kinetics of both dyes is better described by the pseudo-second order kinetic model. The current study provides crucial information regarding the use of waste biomass as an absorbent for the removal of cationic and anionic dyes from aqueous solutions. Most absorption studies use batch systems with a single dye. Effluents that contain dyes are made up of multiple dyes as well as contaminants like metal ions and surfactants.

## Data Availability

All data generated or analyzed during this study are included in this published article.
